# Supplementary material for: Delineation of G Protein-Coupled Receptor Kinase Phosphorylation Sites within the D1 Dopamine Receptor and Their Roles in Modulating β-Arrestin Binding and Activation
Source: Int J Mol Sci. 2023 Apr 1;24(7):6599. doi: 10.3390/ijms24076599 (PMC10095280; doi:10.3390/ijms24076599)
Supplement: Supplementary file 1 [file ijms-24-06599-s001.zip › ijms-2296992-supplementary.pdf]

SUPPLEMENTARY MATERIALS

Table S1. [^{3}H] SCH 23390 saturation binding data for selected D1R constructs used in this study

**D1 Receptor**	**Bmax mean ± SEM (fmol/mg)[1]**	**K_d mean ± SEM (nM)[2]**
WT	1880 ± 650	0.6 ± 0.1
T_0	1270 ± 280	0.7 ± 0.2
T_1	1240 ± 260	0.6 ± 0.1
T_2	3960 ± 910	1.3 ± 0.3
T_3	3040 ± 270	1.2 ± 0.2
S13-20	2620 ± 320	1.1 ± 0.2
Tail Total	940 ± 200*	0.6 ± 0.1
S234	1900 ± 730	0.5 ± 0.2
Rev T5S6	1890 ± 730	1.1 ± 0.3
T5V/S6A	2800 ± 730	1.4 ± 0.4
T_1 + T5V/S6A	2000 ± 640	1.0 ± 0.3
GRK-null	800 ± 120*	0.6 ± 0.03
PKC-null	2050 ± 700	0.8 ± 0.1
PKC-null + A259S	2100 ± 300	1.1 ± 0.2

[1] Bmax values are expressed as mean ± SEM of at least three experiments performed in triplicate. Statistical comparisons between WT and mutant parameters were made using a one-way ANOVA with Dunnett's post-hoc comparison. *$p < 0.05$ vs. WT.

[2] K_d values are expressed as mean ± SEM of at least three experiments performed in triplicate. Statistical comparisons between WT and mutant parameters were made using a one-way ANOVA with Dunnett's post-hoc comparison. For all mutants, $p > 0.05$ vs. WT.

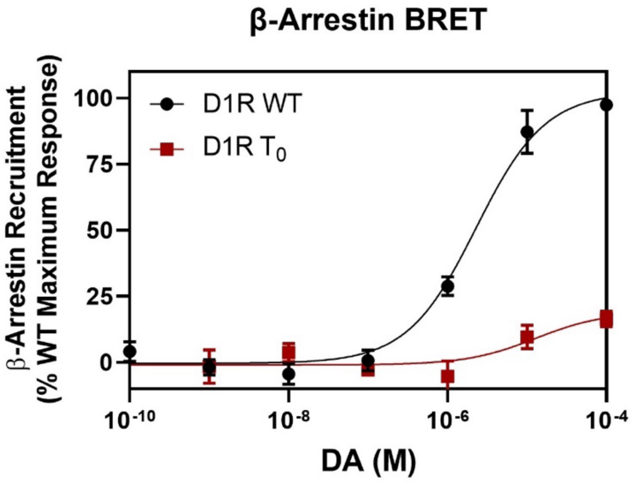

Figure S1. β-arrestin recruitment is severely impaired by the D1R T_0 C-terminal truncation in an orthogonal assay of β-arrestin recruitment. Direct D1R-β-arrestin recruitment BRET assays were performed as described in *Methods*. D1R WT EC_{50} = 2.0 ± 0.4 μM, Emax = 100%; D1R T_0 EC_{50} = undefined, Emax = 13 ± 6.5%***. Data are expressed as a percentage of the maximum WT response to DA and are shown as mean ± SEM of three experiments each performed in triplicate. Statistical comparisons between WT and mutant parameters were made using a t-test: ***$p < 0.001$.

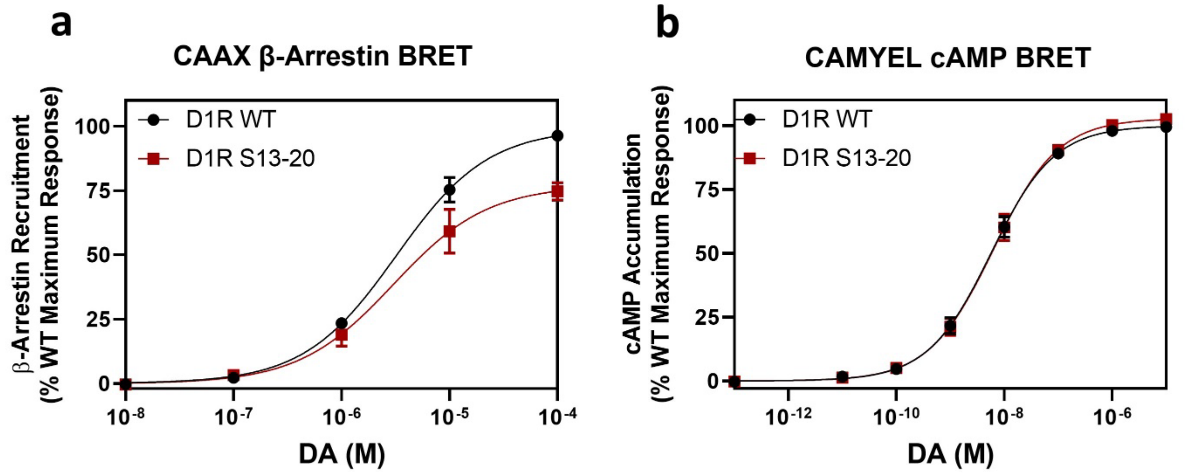

Figure S2. β-arrestin recruitment is impaired by simultaneous mutation of the last eight serines and threonines in the distal C-terminus of the receptor (D1R S13-20). CAAX β-arrestin recruitment and CAMYEL cAMP accumulation assays were performed as described in *Methods*. Data are expressed as a percentage of the maximum D1R WT DA response in each experiment and are displayed as mean ± SEM of at least three experiments, performed in triplicate. **a**) D1R WT EC_{50} = 3.4 ± 0.4 μM, Emax = 100%; D1R S13-20 EC_{50} = 3.7 ± 1.1 μM, Emax = 78 ± 1.7%***. **b**) D1R WT EC_{50} = 6.1 ± 1.5 nM, Emax = 100%; D1R S13-20 EC_{50} = 6.8 nM ± 2.0 nM, Emax = 103% ±

0.6*. Statistical comparisons between WT and mutant parameters were made using a t-test: *$p < 0.05$, ***$p < 0.001$.

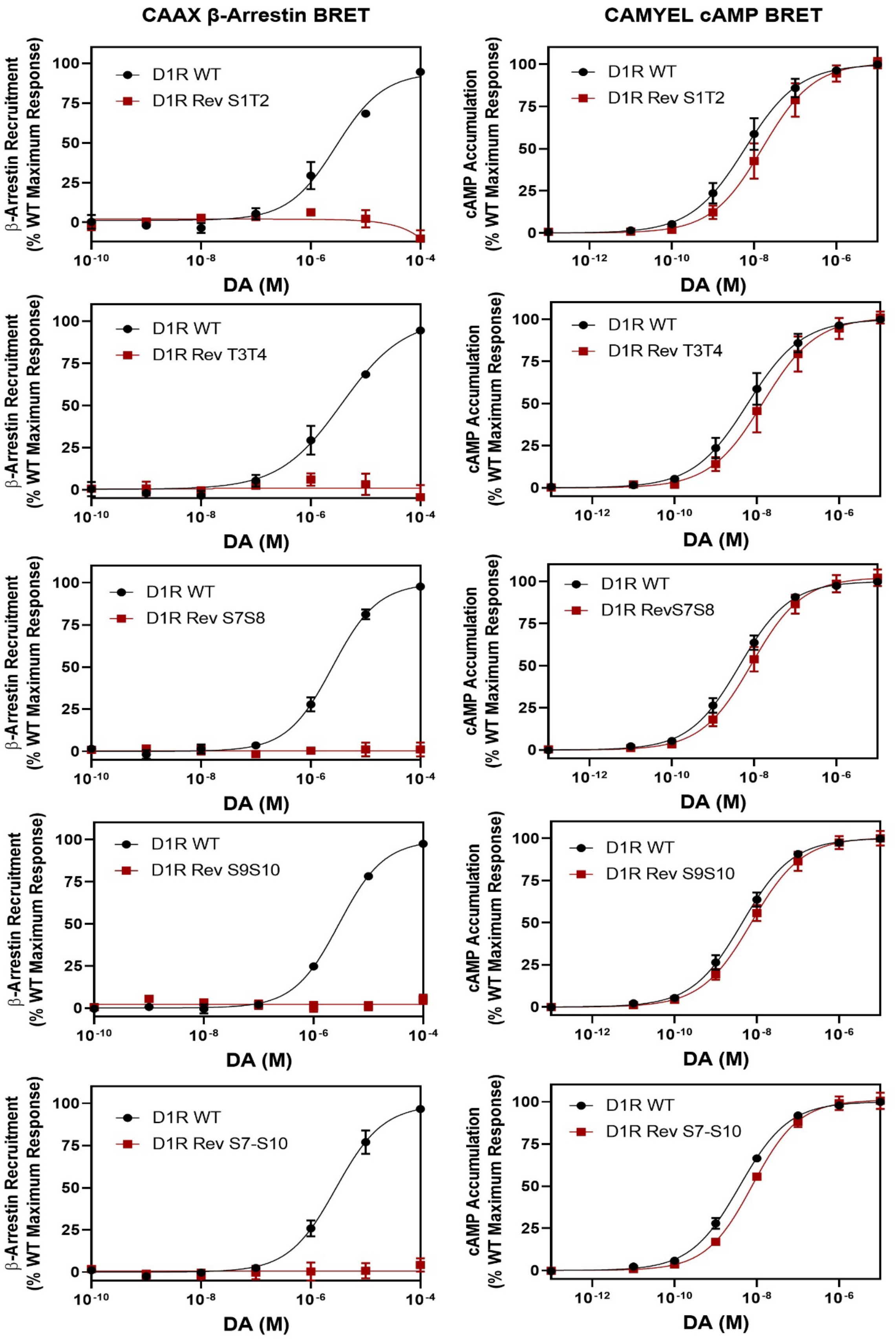
CAAX β-Arrestin BRET
CAMYEL cAMP BRET
β-Arrestin Recruitment (% WT Maximum Response)
cAMP Accumulation (% WT Maximum Response)
DA (M)
D1R WT
D1R Rev S1T2
D1R Rev T3T4
D1R Rev S7S8
D1R RevS7S8
D1R Rev S9S10
D1R Rev S7-S10

Figure S3. The indicated D1R reverse mutants in the proximal C-terminus are unable to recruit β-arrestin with DA stimulation while G-protein signaling is unaffected. CAAX β-arrestin recruitment (left panels) and CAMYEL cAMP accumulation assays (right panels) were performed as described in *Methods*. **Left panels** (from top): D1R WT EC_{50} = 3.6 μM ± 1.1 μM, Emax = 100%; D1R Rev S1T2 EC_{50} > 100 μM, Emax = not defined. D1R WT EC_{50} = 3.6 ± 1.1 μM, Emax = 100%; D1R Rev T3T4 EC_{50} > 100 μM, Emax = not defined. D1R WT EC_{50} = 2.5 ± 0.4 μM, Emax = 100%; D1R Rev S7S8 EC_{50} > 100 μM, Emax = not defined. D1R WT EC_{50} = 2.9 ± 0.3 μM, Emax = 100%; D1R Rev S9S10 EC_{50} > 100 μM, Emax = not defined. D1R WT EC_{50} = 3.3 ± 1.0 μM, Emax = 100%; D1R Rev S7-S10 EC_{50} > 100 μM, Emax = not defined. **Right panels** (from top): D1R WT EC_{50} = 8.8 ± 5.1 nM, Emax = 100%; D1R Rev S1T2 EC_{50} = 26 ± 17 nM, Emax = 101 ± 3.2%. D1R WT EC_{50} = 8.8 ± 5.1 nM, Emax = 100%; D1R Rev T3T4 EC_{50} = 26 ± 19 nM, Emax = 101 ± 3.0%. D1R WT EC_{50} = 4.8 ± 1.4 nM, Emax = 100%; D1R Rev S7S8 EC_{50} = 9.9 ± 3.4 nM, Emax = 102 ± 5.0%. D1R WT EC_{50} = 4.8 ± 1.4 nM, Emax = 100%; D1R Rev S9S10 EC_{50} = 7.8 ± 1.7 nM, Emax = 100 ± 4.2%. D1R WT EC_{50} = 3.9 ± 0.5 nM, Emax = 100%; D1R Rev S7-S10 EC_{50} = 8.0 ± 1.3 nM*, Emax = 101 ± 4.8%. Data are expressed as a percentage of the maximum D1R WT response to DA and are shown as mean ± SEM of at least three experiments each performed in triplicate. Statistical comparisons between D1R WT and mutant parameters in the CAMYEL cAMP accumulation assays made using a t-test: *$p < 0.05$.

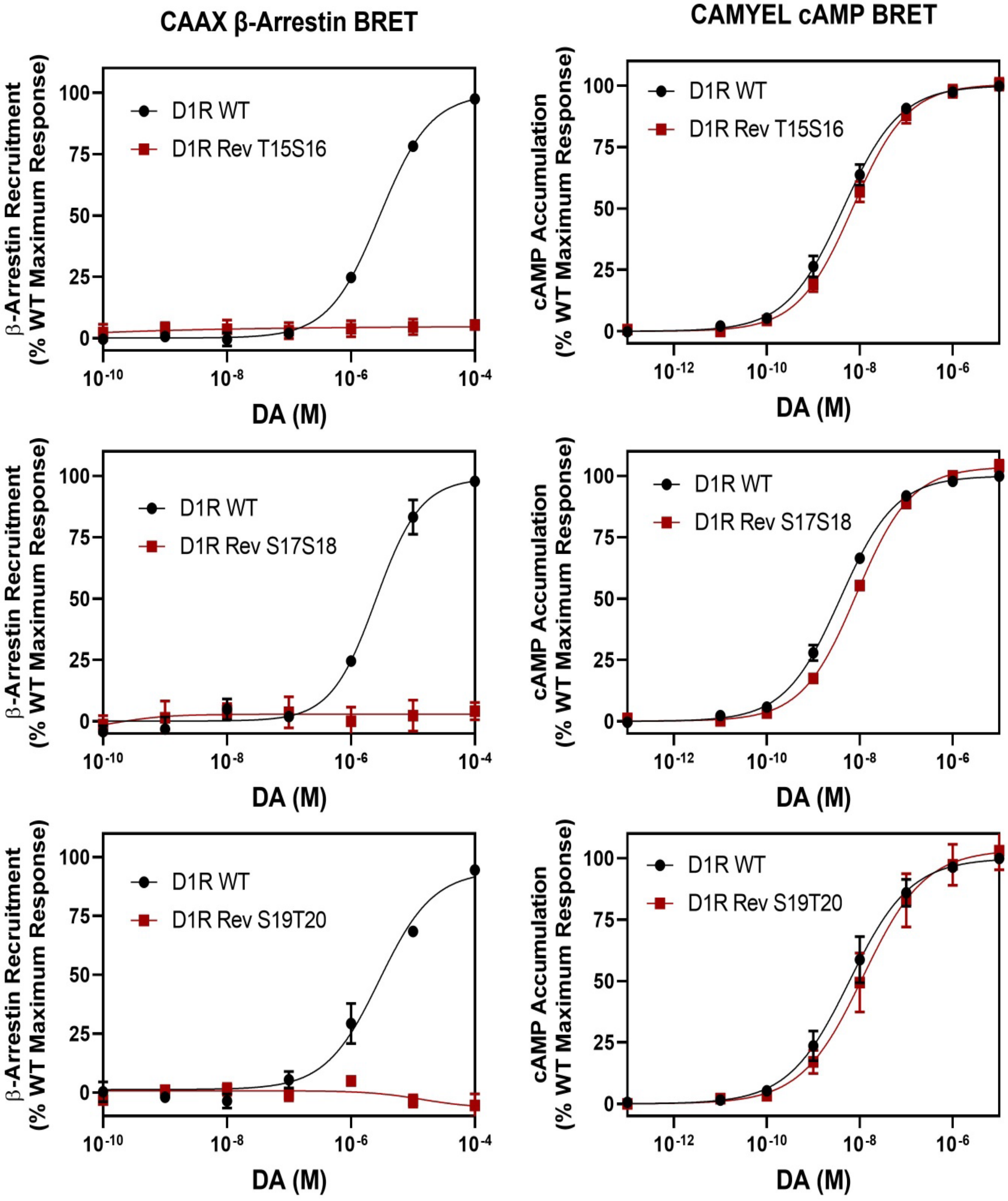
CAAX β-Arrestin BRET
CAMYEL cAMP BRET
β-Arrestin Recruitment (% WT Maximum Response)
cAMP Accumulation (% WT Maximum Response)
DA (M)
D1R WT
D1R Rev T15S16
D1R Rev S17S18
D1R Rev S19T20

Figure S4. The indicated D1R reverse mutants in the distal C-terminus are unable to recruit β-arrestin upon DA stimulation while G-protein signaling is unaffected. CAAX β-arrestin recruitment (left panels) and CAMYEL cAMP accumulation assays (right panels) were performed as described in *Methods*. **Left panels** (from top): D1R WT EC_{50} = 2.9 ± 0.2 μM, Emax = 100%; D1R Rev T15S16 EC_{50} > 100 μM, Emax = not defined. D1R WT EC_{50} = 2.6 ± 0.3 μM, Emax = 100%; D1R Rev S17S18 EC_{50} > = 100 μM, Emax not defined. D1R WT EC_{50} = 3.6 ± 1.1 μM, Emax = 100%; D1R Rev S19T20 EC_{50} > 100 μM, Emax = not defined. **Right panels** (from top): D1R WT EC_{50} = 4.8 ± 1.4 nM, Emax = 100%; D1R Rev T15S16 EC_{50} = 7.5 ± 1.8 nM, Emax = 101 ± 2.5%. D1R WT EC_{50} = 3.9 ± 0.5 nM, Emax = 100%; D1R Rev S17S18 EC_{50} = 8.7 ± 1.2 nM*, Emax = 104 ± 2.0%. D1R WT EC_{50} = 8.8 ± 5.1 nM, Emax = 100%; D1R Rev S19T20 EC_{50} = 18 ± 11 nM, Emax = 103 ± 7.3%. Data are expressed as a percentage of the maximum D1R WT response to DA and are shown as means ± standard error of the mean (SEM) of at least three experiments performed in triplicate. Statistical comparisons between D1R WT and mutant parameters in the CAMYEL cAMP accumulation assays were made using a t-test: *$p < 0.05$.
